# The Correlation Among the Immunoglobulin G Synthesis Rate, IgG Index and Albumin Quotient in Guillain-Barré Syndrome and Chronic Inflammatory Demyelinating Polyradiculoneuropathy: A Retrospective Case–Control Study

**DOI:** 10.3389/fneur.2021.746186

**Published:** 2021-12-17

**Authors:** Yu Tu, Xuan Gong, Yuanyuan Zhang, Jiewei Peng, Wenyan Zhuo, Xueying Yu

**Affiliations:** ^1^Zhuhai People's Hospital (Zhuhai Hospital Affiliated With Jinan University), Jinan University, Zhuhai, China; ^2^Beijing Tiantan Hospital, Capital Medical University, Beijing, China

**Keywords:** Guillain-Barré syndrome, chronic inflammatory demyelinating polyradiculoneuropathy, intrathecal IgG synthesis rate, quotient albumin, IgG index

## Abstract

**Background:** The immunoglobulin G synthesis rate (IgG SR) and immunoglobulin G (IgG) index are indicators of abnormal intrathecal humoural immune responses, and the albumin quotient (QALB) is an indicator used to evaluate the completeness of the blood-cerebrospinal fluid barrier (BCB). No systematic reports regarding differences in Guillain-Barré syndrome (GBS) and chronic inflammatory demyelinating polyradiculoneuropathy (CIDP) are available. We assessed differences in the IgG SR, IgG index and QALB between GBS and CIDP patients in a Chinese cohort.

**Methods:** A total of 234 patients were retrospectively enrolled in this study, and 167 clinically confirmed GBS and CIDP patients were selected. Meanwhile, 67 non-GBS and non-CIDP patients requiring cerebrospinal fluid (CSF) examination were enrolled as the control group. The IgG SR, IgG index and QALB were calculated using formulas. The relevant clinical data were subjected to statistical analysis.

**Results:** Among the GBS and CIDP study groups and the control group, the QALB had the highest positive rate (80.00%) in the CIDP group (*P* < 0.01). The QALB stratification analysis showed that the ranges of 10 < QALB ≤ 30 were dominant in the GBS and CIDP groups, and the positive rate of CIDP was higher than that of GBS. Furthermore, a QALB ≤ 7 was dominant in the control group, and a QALB > 30 was dominant in the CIDP group. In receiver operating characteristic (ROC) curve analysis with the CIDP group as the trial group and the GBS group as the control group, the differences in the QALB were statistically significant (*P* < 0.01). To achieve a high specificity of 98~99%, the diagnostic cut-off value for the QALB was above 57.37 (sensitivity: 9.33%) or below 0.60 (sensitivity: 4.35%). Multivariate logistic regression analysis showed that the CIDP patients had a QALB higher than 57.37, and compared with that in the GBS patients, the difference in the QALB was statistically significant (*P* < 0.01).

**Conclusion:** QALB elevation was associated with CIDP, while QALB values above 57.37 or below 0.60 had high specificity in differentiating between GBS and CIDP. In CIDP, the BCB is generally moderately to severely damaged; in GBS, the BCB is generally moderately damaged.

## Introduction

Guillain-Barré syndrome (GBS) is an immune-mediated demyelinating disease of the peripheral nervous system (PNS) and spinal root and is characterized by the rapid progression of symmetric weakness, disappearance of tendon reflexes, electrophysiological characteristics of demyelination, dysfunction of the blood-cerebrospinal fluid barrier (BCB) and normal cell counts. Chronic inflammatory demyelinating polyradiculoneuropathy (CIDP) is a chronic autoimmune disease of the PNS that shares many common immunopathological changes with GBS (1).

Proteins produced only outside the central nervous system (CNS), such as albumin (ALB), cannot easily cross an intact BCB. However, when the BCB is damaged by CNS diseases, such proteins can cross the blood-brain barrier (BBB) and enter the cerebrospinal fluid (CSF). The CSF/serum albumin quotient (QALB) is currently the indicator most frequently used to evaluate BCB permeability (2).

The sources of immunoglobulin (Ig) in CSF are as follows: (1) blood diffusion through the BCB and (2) local synthesis in the CNS. The determination of the amount of locally synthesized Ig in the CNS, i.e., Ig with components that are not from blood, is essential (3). Intrathecal Ig synthesis occurs in different CNS diseases. Since intrathecal immunoglobulin G (IgG) is the final product of lymphocytic infiltration, intrathecal IgG synthesis can be considered a specific indicator of intrathecal inflammatory lesions (4). The IgG synthesis rate (IgG SR) and IgG index are important indicators of abnormal intrathecal humoural immune responses.

This study compared the differences in the IgG SR, IgG index and QALB between GBS and CIDP with the aim to determine whether the combined detection of IgG SR, IgG index and QALB could contribute to the diagnosis, treatment and prognosis of peripheral nerve demyelinating disease.

## Materials and Methods

### Research Subjects

#### GBS and CIDP Groups

Routine cerebrospinal fluid, cerebrospinal fluid biochemistry and IgG SR for GBS and CIDP patients in Beijing, China, from October 2014 to April 2020: A retrospective study. The inclusion criteria included (1) a diagnosis of GBS or CIDP at Beijing Tiantan Hospital, Capital Medical University according to the Guillain–Barré and Miller-Fisher syndromes—new diagnostic classification (5) and the European Federation of Neurological Societies/Peripheral Nerve Society Guidelines for the management of chronic inflammatory demyelinating polyradiculoneuropathy (6); (2) an age ranging from 18 to 83 years; (3) complete clinical outcome data, including sex, age, CSF examinations, IgG SR, additional blood biochemistry, thyroid antibody, autoimmune antibody spectrum and other examinations; and (4) Hughes Functional Grading Scale (HFGS) score ≤ 4 at the peak of the disease in the GBS and CIDP groups. This retrospective study was approved by our institutional ethics committee, and informed consent was waived.

The exclusion criteria included (1) transient ischaemic attack, stroke, subarachnoid hemorrhage, or encephalitis as the discharge diagnosis; (2) a history of malignant tumors, severe heart, liver and kidney diseases, blood system diseases, and serious infectious diseases; (3) the presence of disabilities and sequelae caused by the patient's neurological diseases; (4) the definite presence of rheumatic diseases; and (5) ventilator-assisted ventilation or death due to GBS or CIDP.

#### Control Group

Non-GBS and non-CIDP patients hospitalized between October 2014 and April 2020 who required CSF examination were enrolled as the control group. The patients suffered from various conditions, such as headaches, multiple sclerosis, leukodystrophy and minor strokes.

### Research Methods

#### Grouping Methods

In total, 234 patients were retrospectively enrolled. The GBS group (92 cases) included 59 cases of classical acute inflammatory demyelinating polyneuropathy, 16 cases of acute motor axonal neuropathy (AMAN), four cases of acute motor-sensory axonal neuropathy, 10 cases of Miller-Fisher syndrome and three cases of acute sensory neuropathy. The CIDP group (75 cases) included 63 cases of typical CIDP, one case of pure motor CIDP and 11 cases of multifocal acquired demyelinating sensory and motor function (MADSAM). Two acute-onset CIDP (A-CIDP) patients were identified through dynamic patient follow-up and review. The control group contained 67 patients, all of whom underwent cerebrospinal fluid examinations.

#### Cerebrospinal Fluid Index Detection and Blood Biochemistry Detection

A venous blood sample was drawn from the cubital vein in the morning within 48 h of admission. The blood samples were stored for 30 min at room temperature and then centrifuged at 3,000 rpm for 10 min in a Hettich centrifuge device. The CSF examination was performed within 2–5 days after admission, and the data from first examination were used if multiple CSF examinations were conducted. The IgG and albumin contents in the serum and CSF samples were determined by rate-scattering turbidimetry using a Siemens BNII automatic protein analyser.

#### Reference Standard Criteria for the Data

(1) QALB: We calculated QALB to determine BBB integrity as follows: [CSF albumin (mg/ml): serum albumin (mg/ml)] × 1000 (2). The calibration standards were prepared as follows: Normal: QALB ≤ 7; mild impairment: 7 < QALB ≤ 10; moderate impairment: 10 < QALB ≤ 30; severe impairment: QALB > 30. (2) QIgG: QIgG was calculated as follows: QIgG = [Cerebrospinal fluid IgG (C-IgG, mg/ml) / Serum IgG (S-IgG, mg/ml)] × 1000. (3) IgG index: The IgG index is defined as the CSF IgG to CSF albumin ratio compared to the serum IgG to serum albumin ratio (7). An IgG index >0.7 indicates intrathecal IgG synthesis in the CSF. (4) IgG SR: The IgG SR was used to determine the occurrence of intrathecal synthesis and was calculated as follows: [(IgG CSF-IgG Serum/369) – (ALB CSF-ALB Serum/230) × (IgG Serum/ALB Serum) × 0.43] × 5 (8). The reference range for IgG SR in our hospital is (−10–10), and >10 is regarded as intrathecal synthesis.

#### Statistical Methods

SPSS statistical software 24.0 was used for the data analysis. The measurement data exhibiting a normal distribution are described as the mean ± standard deviation. Data that did not conform to a normal distribution were compared using the Kruskal-Wallis H test and are expressed as the median, 25th percentile and 75th percentile. The count data are expressed as a percentage (%). The differences between two groups were compared using a chi-squared test. A receiver operating characteristic (ROC) curve analysis was performed using MedCalc 19.1 to evaluate the potential use of the IgG SR, IgG index, QALB and QIgG to predict the GBS and CIDP groups. Indicators with predictive ability were extracted from ROC curve analysis. The cut-off point corresponding to the specificity of the predictive index (98–99%) was used as the standard. Patients with values beyond the cut-off value and those in the control group were analyzed by a binary logistic regression to determine the baseline features of the 1–2% of patients with levels above the cut-off who were considered to have the most severe disease presentation. A linear regression analysis was used to analyse the correlation between the IgG SR and QALB. *P* < 0.05 was considered indicative of a statistically significant difference.

## Results

### Study Participants and Baseline Characteristics

The GBS group consisted of 92 patients (44 females and 48 males, average age 48.30 ± 16.72 years). The CIDP group consisted of 75 patients (23 females and 52 males, average age 50.92 ± 13.26 years). The control group consisted of 67 patients (41 females and 26 males, average age 35.49 ± 12.16 years). The GBS and CIDP patients were older than the control patients (*P* < 0.01), while age and the HFGS score did not significantly differ between the GBS and CIDP groups (*P* > 0.05). Regarding sex, significantly more females were included in the GBS and control groups than in the CIDP group (*P* < 0.05) (Table 1).

**Table 1:** Comparison of the clinical data in the GBS, CIDP and control groups x ± s.

**Variable**	**GBS group (*n* = 92)**	**CIDP group (*n* = 75)**	**Control group (*n* = 67)**	***P* values**
Age (years)	48.30 ± 16.72^Δ^	50.92 ± 13.26^Δ^	35.49 ± 12.16	<0.001
Females *n* (%)	44 (47.83)	23 (30.67)^*Δ^	41 (61.19)	0.001
HFGS	3.71 ± 0.51	3.81 ± 0.39	-	-

Compared to the GBS group, *P < 0.05. Compared to the control group, ^Δ^P < 0.01.

### Comparison of Blood and Cerebrospinal Fluid Indexes in the GBS, CIDP and Control Groups

Cerebrospinal fluid-Protein (C-Pro), Cerebrospinal fluid-Albumin (C-ALB), C-IgG, QALB and QIgG were higher in the GBS and CIDP groups than in the control group, while Cerebrospinal fluid-white blood cell (C-WBC) (*P* < 0.05), S-ALB and the IgG index (*P* < 0.01) in the GBS and CIDP groups were lower than those in the control group. The S-IgG in the GBS group was higher than that in the control group (*P* < 0.01).

The CIDP group had higher levels of C-Pro (*P* < 0.05), C-ALB, QALB and QIgG than the GBS group (*P* < 0.01). The Cerebrospinal fluid-total cell (C-TC) levels (*P* < 0.05), S-IgG (*P* < 0.01) in the CIDP group were lower than those in the GBS group. The differences in the C-WBC, IgG SR, S-ALB, C-IgG and IgG index between the two groups did not reach statistical significance (*P* > 0.05) (Table 2).

**Table 2:** Comparison of blood and cerebrospinal fluid indexes in the GBS, CIDP and control groups *M (P25–P75)*.

**Variable**	**GBS group (*n* = 92)**	**CIDP group (*n* = 75)**	**Control group (*n* = 67)**	***P* values**
C-WBC (/UL)	4.00 (2.00–7.00)^#^	3.00 (1.00–4.00)^#^	6.00 (3.00–10.00)	<0.001
C-TC (/UL)	103.00 (4.00–203.75)	7.00 (2.00–105.50)*	12.00 (5.00–106.00)	0.040
C-Pro (mg/dL)	57.61 (37.92–100.30)^#^	90.40 (53.81–125.40)*^#^	30.00 (24.31–42.52)	<0.001
IgG SR	6.44 (0.75–23.65)	11.53 (3.03–22.16)	6.33 (2.72–13.97)	0.237
C-ALB (mg/ml)	0.34 (0.19–0.63)^#^	0.62 (0.33–1.00)**^#^	0.21 (0.14–0.27)	<0.001
S-ALB (mg/ml)	39.25 (35.23–42.85)^#^	40.50 (35.40–42.90)^#^	42.90 (39.60–46.70)	<0.001
C-IgG (mg/ml)	0.07 (0.04–0.14)^#^	0.08 (0.05–0.16)^#^	0.04 (0.03–0.06)	<0.001
S-IgG (mg/ml)	11.85 (10.23–14.85)^#^	10.80 (9.16–12.00)**	10.40 (9.08–12.20)	<0.001
QALB	8.86 (4.77–17.60)^#^	15.15 (7.69–28.57)**^#^	4.62 (3.47–6.22)	<0.001
QIgG	5.42 (2.74–11.84)^#^	8.09 (4.74–14.00)**^#^	3.64 (2.33–4.84)	<0.001
IgG index	0.54 (0.46–0.65)^#^	0.56 (0.49–0.64)^#^	0.66 (0.55–1.09)	<0.001

Compared to the GBS group (*P < 0.05, **P < 0.01). Compared to the control group (^#^P < 0.01).

### Comparison of the Positive IgG SR, QALB and IgG Index Rates in the GBS, CIDP and Control Groups

**Figure 1:**
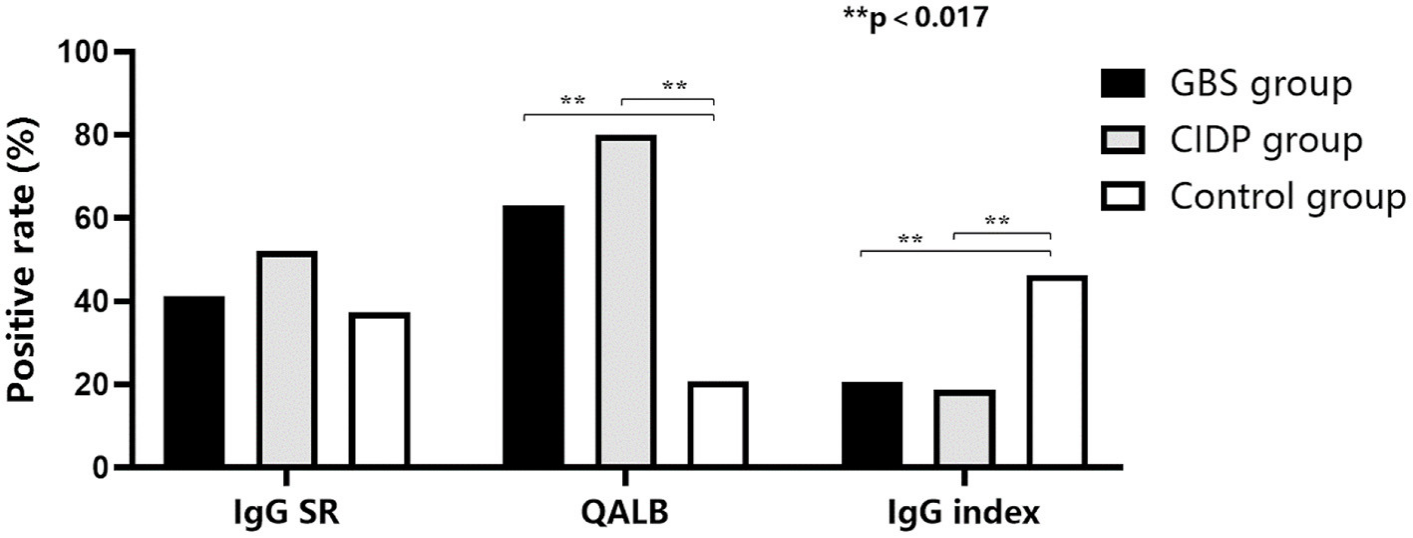
Comparison of the positive IgG SR, QALB and IgG index rates in the GBS, CIDP and control groups. The positive QALB rate in the GBS and CIDP groups was significantly higher than that in the control group (*P* < 0.017). The positive IgG index rate in the GBS and CIDP groups was significantly lower than that in the control group (*P* < 0.017).

**Figure 2:**
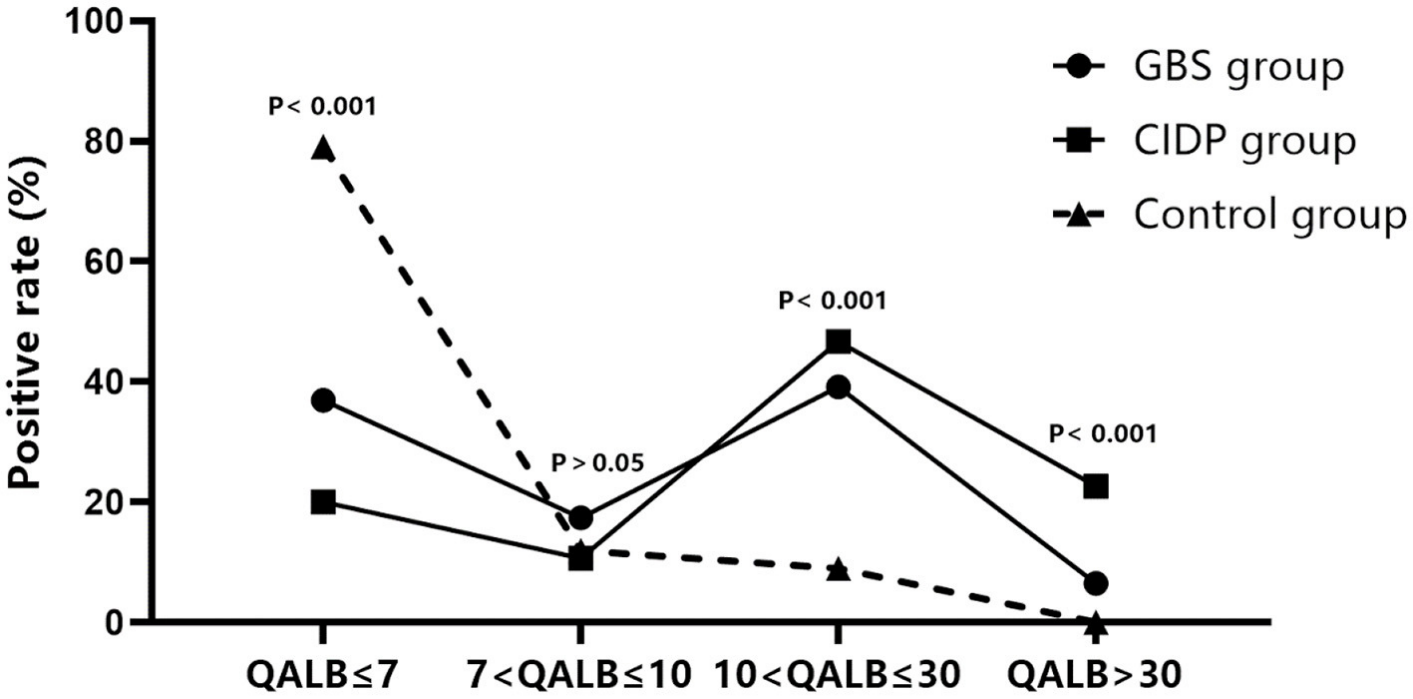
QALB stratification analysis in the GBS, CIDP and control groups. QALB stratification analysis showed that the ranges of 10 < QALB ≤ 30 were dominant in the GBS and CIDP groups, and the positive rate of CIDP was higher than that of GBS. Furthermore, a QALB ≤ 7 was dominant in the control group, and a QALB > 30 was dominant in the CIDP group.

Normal IgG SR, QALB and IgG index values were used as the reference standards, and positive cases were defined as those exceeding the upper limit. IgG SR: The positive case numbers and positivity rates were 38 (41.30%) in the GBS group, 39 (52.00%) in the CIDP group and 25 (37.31%) in the control group. No statistically significant difference was found among the three groups (*P* > 0.05). QALB: The positive case numbers and positivity rates were 58 (63.04%) in the GBS group, 60 (80.00%) in the CIDP group and 14 (20.90%) in the control group. A statistically significant difference was identified among the three groups (*P* < 0.001). According to the results, the positive QALB rates in the GBS and CIDP groups were significantly higher than that in the control group. QALB stratification analysis showed that the ranges of 10 < QALB ≤ 30 were dominant in the GBS and CIDP groups, and the positive rate of CIDP was higher than that of GBS. Furthermore, a QALB ≤ 7 and an IgG index > 0.7 were dominant in the control group, and a QALB > 30 was dominant in the CIDP group, which may indicate that in CIDP, damage to the BCB is mainly moderate to severe. The GBS group showed mainly moderate injuries to the BCB. IgG index: The positive case numbers and positivity rates were 19 (20.65%) in the GBS group, 14 (18.67%) in the CIDP group and 31 (46.27%) in the control group. A statistically significant difference was observed among the three groups (*P* < 0.001), and the positive IgG index rates in the GBS and CIDP groups were significantly lower than that in the control group (Table 3 and Figures 1, 2).

**Table 3:** Comparison of the IgG SR, QALB, and IgG index positive rates in the GBS, CIDP and control groups.

**Variable**	**GBS group (*n* = 92)**	**CIDP group (*n* = 75)**	**Control group (*n* = 67)**	***P* values**
**QALB**, ***n*** **(%)**				
QALB ≤ 7 (normal)	34 (36.96)^#^	15 (20.00)^#^	53 (79.10)	<0.001
7 < QALB ≤ 10	16 (17.39)	8 (10.67)	8 (11.94)	0.402
10 < QALB ≤ 30	36 (39.13)^#^	35 (46.67)^#^	6 (8.96)	<0.001
QALB > 30	6 (6.52)	17 (22.67)**^#^	0 (0.00)	<0.001
Total (abnormal)	58 (63.04)^#^	60 (80.00)^#^	14 (20.90)	<0.001
IgG SR > 10, *n* (%)	38 (41.30)	39 (52.00)	25 (37.31)	0.180
IgG index > 0.7, *n* (%)	19 (20.65)^#^	14 (18.67)^#^	31 (46.27)	<0.001

Compared to the GBS group (**P < 0.017). Compared to the control group (^#^P < 0.017).

The QALB, IgG SR and IgG index in GBS and CIDP subtypes were analyzed. The QALB in the AMAN subtype was lower than that in the typical CIDP subtype, *P* (Adjusted) < 0.05. Among the patients with moderate-to-severe increases in the QALB (QALB >10), the typical CIDP subtype was the most common (74.60%). No statistical differences in the IgG SR and IgG index were found between the GBS and CIDP subtypes (*P* > 0.05) (Table 4).

**Table 4:** Correlation analysis between QALB and GBS, CIDP subtypes *M (P25–P75)*.

	**QALB ≤ 10 *n* (%)**	**QALB >10 *n* (%)**	**QALB**	**IgG SR**	**IgG index**
**GBS group**					
AIDP (*n* = 59)	27 (45.76)	32 (54.24)	10.33 (4.90–19.44)	8.93 (0.57–22.83)	0.55 (0.46–0.64)
MFS (*n* = 10)	7 (70.00)	3 (30.00)	7.99 (6.27–20.90)	1.67 (-0.41–50.27)	0.50 (0.44–0.69)
AMAN (*n* = 16)	11 (68.75)	5 (31.25)	7.49 (3.12–11.29)*	6.47 (0.87–20.15)	0.54 (0.47–1.96)
AMSAN (*n* = 4)	2 (50.00)	2 (50.00)	6.06 (0.56–40.24)	23.35 (2.45–56.12)	2.74 (0.49–5.66)
ASN (*n* = 3)	3 (100.00)	0 (0.00)	6.59 (3.31–9.04)	1.37 (0.50–2.62)	0.49 (0.46–0.50)
**CIDP group**					
Typical CIDP (*n* = 63)	16 (25.40)	47 (74.60)	15.82 (9.86–31.59)	11.04 (2.51–19.44)	0.56 (0.48–0.64)
MADSAM (*n* = 11)	6 (54.55)	5 (45.45)	7.69 (4.78–19.77)	12.19 (3.40–25.55)	0.64 (0.55–3.12)
Pure motor CIDP (*n* = 1)	1 (100.00)	0 (0.00)	-	-	-
*χ^2^*	21.32	21.83	7.17	10.83	
*P* value	0.003	0.003	0.412	0.146	

Compared to the Typical CIDP, *P (Adjusted) < 0.05.

### Analyses of the ROC Curves of the IgG SR, IgG Index, QALB and QIgG in the GBS and CIDP Groups

**Figure 3:**
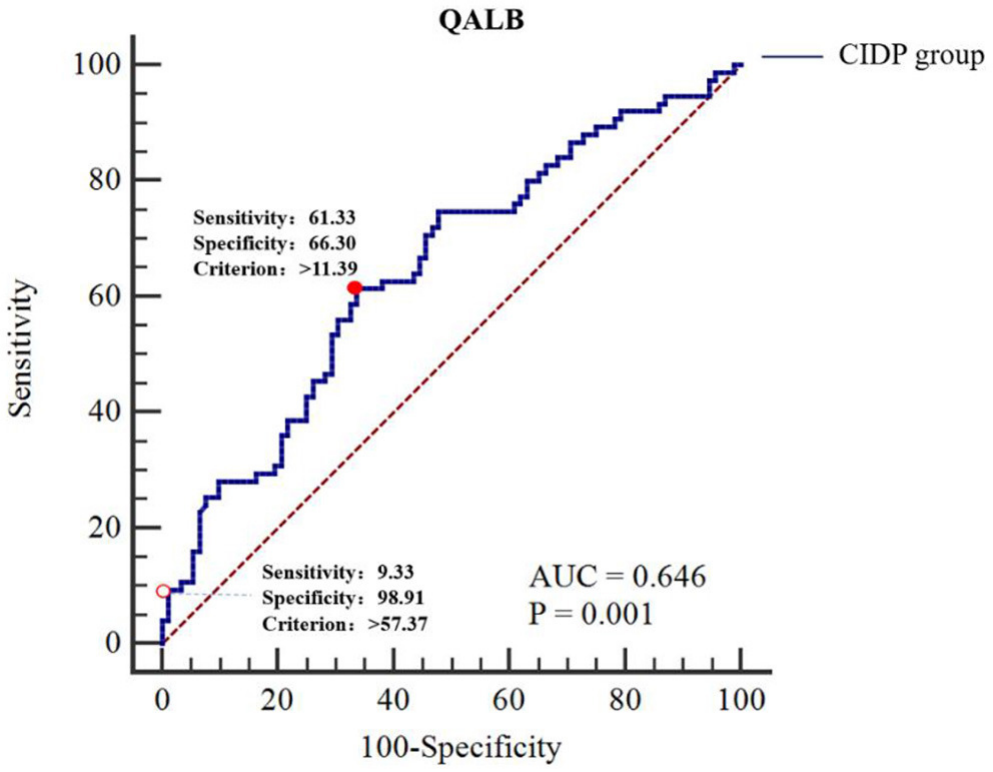
ROC curve analysis of QALB in the GBS and CIDP groups. The GBS group was used as the reference group, and the CIDP group was used as the measurement group. 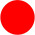 The diagnostic cut-off value corresponds to the maximum point of the Youden index. 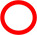 The diagnostic cut-off value corresponds to the maximal specificity.

The ROC curve analyses were performed as follows: the CIDP group was the trial group, and the GBS group was the control group. We found that the differences in QALB and QIgG were statistically significant (*P* < 0.01). The IgG index and IgG SR did not have a statistically significant effect (*P* > 0.05). The cut-off value was determined by the maximum Youden index. The best cut-off value for QALB was 11.39, which achieved a specificity of 66.30% and a sensitivity of 61.33% (95% confidence interval (CI): 0.568–0.718). When the cut-off was > 57.37, QALB had a sensitivity of 9.33% and a specificity of 98.91%; when the cut-off was lower than 0.60, QALB had a sensitivity of 4.35% and a specificity of 98.67% (Table 5 and Figure 3). The best cut-off value for QIgG was 5.39, which achieved a specificity of 50.00% and a sensitivity of 73.33% (95% CI: 0.552–0.703). When the cut-off was >36.74, QIgG had a sensitivity of 9.33% and a specificity of 98.91%; when the cut-off was lower than 1.47, QIgG had a sensitivity of 10.87% and a specificity of 98.67% (Table 5). QALB was accepted as the best indicator for assessing the GBS and CIDP groups.

**Table 5:** Analysis of the ROC curves of the IgG SR, IgG index, QALB and QIgG in the GBS and CIDP groups.

**Variable**	**AUC**	**SE**	**95% CI**	**Youden index**	***P* values**	**Criterion**	**Specificity (%)**	**Sensitivity (%)**
IgG SR	0.553	0.045	0.474–0.630	0.181	0.234	-	-	-
IgG index	0.547	0.045	0.468–0.624	0.155	0.290	-	-	-
QALB	0.646	0.043	0.568–0.718	0.276	0.001**	>11.39^#^	66.30	61.33
						>57.37^Δ^	98.91	9.33
						≤ 0.60^Δ^	98.67	4.35
QIgG	0.630	0.043	0.552–0.703	0.233	0.003**	>5.39^#^	50.00	73.33
						>36.74^Δ^	98.91	9.33
						≤ 1.47^Δ^	98.67	10.87

**P < 0.01. ^#^The diagnostic cut-off value corresponds to the maximum point of the Youden index. ^Δ^The diagnostic cut-off value corresponds to the maximal specificity. In the GBS and CIDP groups, the GBS group was used as the reference group, and the CIDP group was used as the measurement group. The differences in QALB and QIgG between the GBS and CIDP groups were statistically significant, **P < 0.01.

### Logistic Regression Analysis of the Risk Factors for CIDP in Patients With QALB > 57.37 or QIgG > 36.74

In the GBS and CIDP groups, CIDP patients with QALB > 57.37 or QIgG > 36.74 and GBS patients were used as classification-dependent variables, and general patient data, including sex, age, C-WBC, C-TC, QALB, QIgG, IgG SR, IgG index and HFGS, were used as independent variables in the univariate logistic regression analysis. Variables with *P* < 0.05 were included in the multivariate logistic regression analysis. The results of the regression showed that when CIDP patients with QALB > 57.37 were compared with GBS patients, the difference in QALB was statistically significant (*P* < 0.01). When CIDP patients with QIgG > 36.74 were compared with GBS patients, the difference in QIgG was not statistically significant (*P* > 0.05) (Table 6).

**Table 6:** Logistic regression analysis of the risk factors for CIDP in patients with QALB > 57.37 or QIgG > 36.74.

**Risk factors**	**CIDP patients with QALB** **>** **57.37**				**CIDP patients with QIgG** **>** **36.74**			
	**Univariate analysis**		**Multivariate analysis**		**Univariate analysis**		**Multivariate analysis**	
	**OR**	***P* values**	**OR**	***P* values**	**OR**	***P* values**	**OR**	***P* values**
Sex	0.436	0.336	-	-	0.182	0.121	-	-
Age	0.993	0.752	-	-	0.985	0.532	-	-
C-WBC (/UL)	0.992	0.919	-	-	0.974	1.002	-	-
C-TC (/UL)	0.997	0.388	-	-	0.996	0.314	-	-
QALB	1.089	0.002**	1.109	0.004**	1.085	0.002**	0.198	0.973
QIgG	1.118	0.001**	0.978	0.093	1.128	0.001**	225.638	0.967
IgG SR	1.012	0.050	-	-	1.015	0.025*	0.481	0.966
IgG index	1.117	0.609	-	-	1.133	0.557	-	-
HFGS	0.410	0.176	-	-	0.410	0.176	-	-

*P < 0.05, **P < 0.01. Data from CIDP patients with a QALB > 57.37 or QIgG > 36.74 and GBS patients were used as classification-dependent variables, and general patient data, including sex, age, levels of C-WBC, C-TC, QALB and QIgG and IgG SR, IgG index and HFGS values, were used as independent variables in the univariate logistic regression analysis. Variables with P < 0.05 were included in the multivariate logistic regression analysis.

### Linear Correlation Analysis of the IgG SR and QALB in the GBS and CIDP Groups

**Figure 4:**
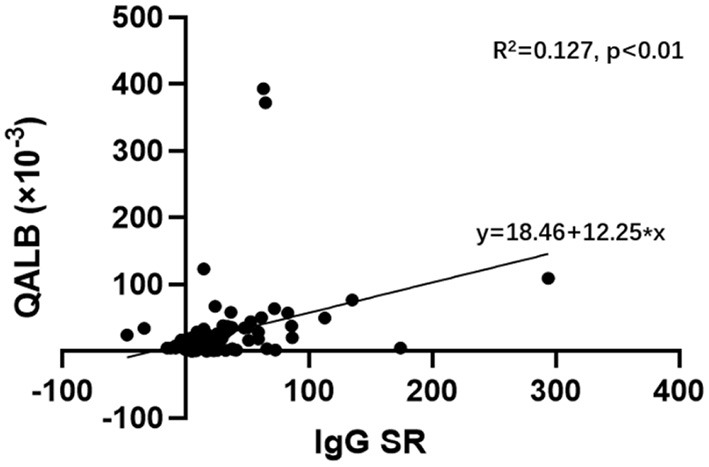
Linear regression analysis of the IgG SR and QALB in the GBS and CIDP groups. Linear regression analysis showed a linear correlation between the IgG SR and QALB (R^2^ = 0.127, *P* < 0.01) in the GBS and CIDP groups.

A linear regression analysis was used to analyse the correlation between the IgG SR and QALB in the GBS and CIDP groups. The results showed a linear correlation between the IgG SR and QALB (R^2^ = 0.127, *P* < 0.01, Figure 4).

## Discussion

### Correlations of the QALB in the GBS, CIDP and Control Groups

Damage to tight junctions in the BCB and dysregulation of transporter proteins increase BCB permeability (9). QIgG and QALB are both indicators used to evaluate the completeness of the BCB (10). CIDP and GBS significantly increased CSF protein and blood-cerebrospinal fluid barrier damage (11). An increase in the QALB was a significant independent predictor of demyelinating GBS independent of age and sex (12).This study showed that the QALB and QIgG were highest in CIDP group, indicating the most severe damage to the BCB, followed by the GBS group, and the lowest value was observed in the control group. Regarding the mechanism responsible for QALB elevation, in addition to the increase in BCB permeability due to damage to the BCB, the decreased CSF flow rate should be considered. Studies have shown that in GBS and CIDP, elevated QALB may be due to a reduced CSF flow rate caused by inflammatory swelling in the spinal roots (13). Related studies investigating rats with experimental autoimmune neuritis have confirmed that a rapid increase in the entry of Ig into the spinal nerve roots is accompanied by the infiltration of T lymphocytes and polymorphonuclear leukocytes in the nervous system (14). In GBS and CIDP, nerve root inflammation changes the permeability of nerves and the walls of blood vessels around nerves, leading to increased leakage. Ig can leak from damaged nerve roots into the CSF, leading to increased QIgG and QALB and finally BCB dysfunction. This mechanism may be promoted in patients with positive serum anti-ganglioside antibodies.

As a widely used biomarker, the QALB helps monitor neuroinflammatory diseases (such as demyelinating diseases). Relevant animal experiments have shown that in active demyelinating lesions, BCB permeability is significantly increased, while in inactive demyelinating lesions, there is very limited or almost no damage to the BCB (15). This study found that the QALB in the CIDP patients was higher than that in the GBS patients; furthermore, compared with the GBS group, in the CIDP group, QALB was an independent risk factor even when QALB >57.37. Relevant studies have shown that elevated serum matrix metalloproteinase-9 (MMP-9) levels are associated with severe GBS but less associated with GBS with mild symptoms (16). MMP-9 levels are significantly increased in CIDP patients (17). MMP-9 mainly originates from T cells and plays an important role in the pathogenesis of BCB damage by regulating the complex intercellular connective structure in brain microvascular endothelial cells 22 (BMECs22) (18). Moreover, MMP-9 and a variety of inflammatory mediators, such as IL-1β, TNF-a and IL-6, form a complex inflammatory network and play a core regulatory role. Prolonged secretion and stimulation of inflammatory mediators may increase MMP-9 levels and BCB permeability (19). Based on previous mechanism studies, the continuous activity of CIDP (i.e., a long disease course) is speculated to increase the concentration of MMP-9 and destruction of the BCB, which is a possible reason for the higher QALB in the CIDP group than in the GBS group.

Analysis of the GBS and CIDP subtypes found that the typical CIDP subtype was the most common among patients with moderate QALB levels and moderate-to-severe QALB levels. Shimizu et al. showed that compared with MADSAM or distal acquired demyelinating symmetric (DADS) patients, typical CIDP patients had a more severely damaged BCB. In serum obtained from typical CIDP patients, the levels of claudin-5 protein and transendothelial electrical resistance (TEER) in peripheral nerve microvascular endothelial cells (PnMECs) were significantly reduced (20, 21). This study further supports previous results.

### Correlations of the IgG SR and IgG Index in the GBS, CIDP and Control Groups

The IgG SR and IgG index are more indicative than the absolute concentration of IgG in CSF and are important indicators for the determination of abnormal intrathecal humoural immune responses. The IgG index can exclude the effect of S-IgG on the C-IgG level and can determine whether C-IgG is the result of serum infiltration or intrathecal synthesis in the CNS. The IgG SR can exclude the impact of serum and BCB factors on the C-IgG level and can determine the level of endogenous IgG in CSF. However, the IgG SR and IgG index are used under restricted conditions. The calculation of the IgG SR assumes that the ability of ALB and IgG to breach the BCB is not related to damage to BCB, implying that the formulas used to calculate the IgG index and IgG SR are only applicable to patients with no or minor damage to the BCB (8). The specificity of the IgG index decreases with increasing BCB permeability. This study found that the IgG index was higher in the control group than in the GBS and CIDP groups. The BCB damage in the control group was minimal; thus, the increase in the IgG index indicates the presence of intrathecal synthesis in the control group, which is considered to be related to the enrolment of multiple sclerosis patients in the control group (22).

Intrathecal synthesis in GBS and CIDP remains controversial. One study found that the response of serum anti-ganglioside in GBS was associated with an increased intrathecal IgG SR, while no such finding was observed in CIDP (23). An intrathecal humoural immune response is rare in our patients with CIDP (11). Studies have shown that mycoplasma pneumoniae IgG antibody synthesized in the sheath was found in the cerebrospinal fluid of patients with GBS (24). Moreover, in GBS and CIDP patients, there is no evidence of intrathecal oligoclonal IgG bands (OBs) in CSF or intrathecal synthesis of anti-ganglioside antibodies; therefore, the use of only the IgG SR to evaluate this group of patients could yield false positive results related to intrathecal IgG synthesis (23). This study showed that the IgG SR was elevated in CIDP, but no difference in the IgG SR was found among CIDP, GBS and control patients. The possible reasons are as follow: (1) CIDP and GBS patients have different degrees of BCB damage. Therefore, the IgG SR and IgG index suggest potential erroneous results in the GBS and CIDP patients. (2) The increase in the IgG SR in CIDP is due to leakage from nerve roots, not the large amount of IgG generated by intrathecal synthesis.

From linear regression analysis, the IgG SR and QALB were linearly positive correlated in the GBS and CIDP groups. Studies have shown that MMP-9 is negatively correlated with the IgG SR in CNS diseases with evident intrathecal synthesis (25). Combined with the aforementioned MMP-9 damage to BCB permeability, we speculated that the QALB should be negatively correlated with the IgG SR, which is not consistent with the conclusion of this study. From a different aspect, the above analysis confirms that the IgG in GBS and CIDP patients is not from intrathecal synthesis, and the false-positive rate of the IgG SR may increase with increasing damage to the BCB.

This study was a retrospective cross-sectional study that did not collect or detect gangliosides in each patient and did not reveal the correlations between gangliosides and the IgG SR, IgG index and QALB in GBS and CIDP. In addition, since this was a cross-sectional study, patients could not be followed to dynamically observe changes in related indicators. Prospective case-control studies and cohort studies should be performed in the future to improve the examination and follow-up of patients and produce more detailed and accurate studies.

In summary, our study indicated that an elevated QALB was associated with CIDP, while a QALB higher than 57.37 or lower than 0.60 had high specificity for differentiating between GBS and CIDP. In CIDP, the BCB was generally moderately to severely damaged; in GBS, the BCB was generally moderately damaged. Future work should attempt to repair damaged BCB early to prevent deterioration and progression in GBS and CIDP.

## Data Availability Statement

The original contributions presented in the study are included in the article/supplementary material, further inquiries can be directed to the corresponding author/s.

## Ethics Statement

The studies involving human participants were reviewed and approved by Beijing Tiantan Hospital Institutional Ethics Committee. Written informed consent for participation was not required for this study in accordance with the national legislation and the institutional requirements.

## Author Contributions

YT managed and collected data and wrote the manuscript. XG researched the data and wrote the manuscript. XY conceived and designed the study and contributed to the manuscript. YZ, JP, and WZ researched the data, contributed to the discussion, and reviewed and edited the manuscript. All authors contributed to the article and approved the submitted version.

## Funding

The present study was supported by the Zhuhai Science and Technology Plan Project (No. 20191208E030034).

## Conflict of Interest

The authors declare that the research was conducted in the absence of any commercial or financial relationships that could be construed as a potential conflict of interest.

## Publisher's Note

All claims expressed in this article are solely those of the authors and do not necessarily represent those of their affiliated organizations, or those of the publisher, the editors and the reviewers. Any product that may be evaluated in this article, or claim that may be made by its manufacturer, is not guaranteed or endorsed by the publisher.
